# Registry-Based Mortality Analysis Reveals a High Proportion of Patient Decrees and Presumed Limitation of Therapy in Severe Geriatric Trauma

**DOI:** 10.3390/jcm9092686

**Published:** 2020-08-19

**Authors:** Cora Rebecca Schindler, Mathias Woschek, René Danilo Verboket, Ramona Sturm, Nicolas Söhling, Ingo Marzi, Philipp Störmann

**Affiliations:** Department of Trauma, Hand and Reconstructive Surgery, University Hospital Frankfurt, 60590 Frankfurt, Germany; mathias.woschek@kgu.de (M.W.); rene.verboket@kgu.de (R.D.V.); ramona.sturm@kgu.de (R.S.); nicolas.soehling@kgu.de (N.S.); marzi@trauma.uni-frankfurt.de (I.M.); philipp.stoermann@kgu.de (P.S.)

**Keywords:** polytrauma, trauma registry, geriatric patients, quality of life, severely injured, patient’s decree, mortality analysis

## Abstract

Background: The treatment of severely injured patients, especially in older age, is complex, and based on strict guidelines. Methods: We conducted a retrospective study by analyzing our internal registry for mortality risk factors in deceased trauma patients. All patients that were admitted to the trauma bay of our level-1-trauma center from 2014 to 2018, and that died during the in-hospital treatment, were included. The aim of this study was to carry out a quality assurance concerning the initial care of severely injured patients. Results: In the 5-year period, 135 trauma patients died. The median (IQR) age was 69 (38–83) years, 71% were male, and the median (IQR) Injury Severity Score (ISS) was 25 (17–34) points. Overall, 41% of the patients suffered from severe traumatic brain injuries (TBI) (AIS_head_ ≥ 4 points). For 12.7%, therapy was finally limited owing to an existing patient’s decree; in 64.9% with an uncertain prognosis, a ‘therapia minima’ was established in consensus with the relatives. Conclusion: Although the mortality rate was primarily related to the severity of the injury, a significant number of deaths were not exclusively due to medical reasons, but also to a self-determined limitation of therapy for severely injured geriatric patients. The conscientious documentation concerning the will of the patient is increasingly important in supporting medical decisions.

## 1. Introduction

Accident-related deaths continue to be among the most frequent causes of death, worldwide and in Germany. The main reasons for death in those patients are typically severe multiple trauma following traffic accidents and (isolated) severe traumatic brain injuries (TBI) after falls, especially in older age [1,2]. The treatment of multiple injured patients is complex, especially due to the presence of injuries in different parts of the body and the time-critical initial treatment [3,4]. The care for severely injured trauma patients has changed during recent decades and is currently based on strict guidelines. Algorithms such as Advanced Trauma Life Support (ATLS^®^) or the European Trauma Course (ETC^®^) are designed to avoid errors and to further standardize the rapid and unpredictable treatment of patients with multiple injuries [5]. Trauma surgery, with its high proportion of geriatric patients, is often confronted with the problem of expanding the therapy for seriously injured patients. In this context, living wills, preventive powers of attorney or legal care can represent important support for the medical decision [6,7].

Quality management (QM) is becoming an increasingly important tool for monitoring the quality of patient care, including the analysis of risk factors, missed injuries or unexpected deaths [8]. According to the World Health Organization (WHO), an estimated 2.6 million people in 150 low and middle-income countries die annually due to incorrect medical treatment [9]. In 2011, 18,800 patients died in German hospitals following preventable adverse events (PAEs) [10]. The majority of errors were caused by faulty systems, processes, and conditions [11]. Worldwide, QM has become a high-priority topic, and in Germany since 2000, a legal obligation [12,13,14]. Medical patient registries are an important source of knowledge on epidemiological issues, quality assurance and short-term statements on the current status of care practice [15,16]. One example is the trauma registry of the German Association of Trauma Surgery (TraumaRegister DGU^®^, TR-DGU^®^), which is one of the largest registries for severely injured patients worldwide [17,18].

The main objective of the present study was a retrospective analysis of our internal registry of trauma patients with regard to the causes of death of severely injured patients who died in hospital, the factors influencing treatment quality and decisions, and avoidable events.

## 2. Experimental Section

We conducted a retrospective study and data analysis, based on the data discussed previously, during our interdisciplinary morbidity and mortality conference. The study is based on the demographic and clinical data of all patients who were admitted to the trauma bay of our Level 1 Trauma Center (TC) between 2014 and 2018 and who died for any reason during their stay in hospital. Throughout the study, the data for each of the deceased patients were analyzed in relation to the injury pattern and clinical data for treatment before and during hospitalization. The aim was to identify factors that influence treatment and patient outcome in order to improve the quality and safety of patient care.

Management of multiple trauma is performed in an interdisciplinary team (trauma surgeons, anesthesiologist, radiologist, nurses) in concordance to the rules of ATLS^®^ [5]. The severity of a patient’s injury was analyzed using the Abbreviated Injury Scale (AIS), the Injury Severity Score (ISS), and the New Injury Severity Score (NISS) [19,20]. To calculate a mortality prognosis RISC II Score (Revised Injury Severity Classification, Version 2) was used [21].

Patient data, which are transmitted to TR-DGU^®^ in pseudonymized form, were simultaneously collected in a central in-house database. This database contains information on demography, injury patterns, comorbidities, preclinical and clinical management, and intensive care progress, as well as significant laboratory findings and outcome data. Relevant information collected in the context of emergency care is recorded by a documentation assistant and immediately entered into the database to ensure the quality of the data. The analysis also included a careful retrospective review of the files of the *n* = 135 deceased patients to supplement relevant clinical information in addition to the registry data.

Our study followed the STROBE guidelines for observational studies (Strengthening the Reporting of Observational Studies in Epidemiology) and the RECORD guidelines for observational studies (Reporting of studies Conducted using Observational Routinely Collected Data) [22,23]. This study was conducted after approval by the Institutional Review Board of the University Hospital of the Goethe University Frankfurt (EV 20–537).

### Statistical Analysis

Continuous variables were presented in median and interquartile range (IQR), or in percentages for categorical variables. Differences in variance between ISS were assessed by the use of Student’s *t*-test. *p* values < 0.05 were considered statistically significant for all tests. All analyses were performed using the Statistical Package for Social Sciences (SPSS for Mac©), version 26 (SPSS Inc., Chicago, IL, USA).

## 3. Results

During the 5-year period, *n* = 2191 patients were admitted to the trauma bay of our TC with suspicion of severe multiple trauma. Of these patients, *n* = 135 (6.2%) patients died. Within the framework of an interdisciplinary quality circle, all 135 cases were evaluated, based on the available data.

**Figure 1:**
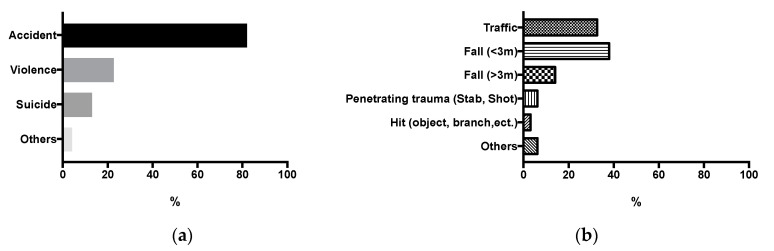
(**a**) Cause of admittance and (**b**) accident details in non-survivors.

As shown in Figure 1, 110 patients were admitted following an accident (81.5%), *n* = 17 (12.6%) were admitted after attempted suicide, *n* = 5 (3.7%) were not classified and *n* = 3 (2.2%) suffered from interpersonal violence. The largest proportion of all injuries was from falls, followed by traffic accidents. A total of *n* = 18 (14.0%) fell from a significant height (>3 m), whereas *n* = 49 (38.0%) suffered a fall from a lower height (<3 m). Among the traffic accident patients, mainly car or truck drivers were afflicted (*n* = 14, 37.8%), followed by pedestrians (*n* = 10, 27.0%), cyclists (*n* = 8, 21.6%) and motorcyclists (*n* = 5, 13.5%).

Table 1 displays the basic patient data. The median (IQR) age of the deceased was 69 (38–83) years and 71.1% were male. The initially calculated median (IQR) ISS taken from the patient charts was 25 (17–34) points. In total, *n* = 110 patients were severely injured with an ISS ≥16 points; 16.3% of the deceased had an ISS ≥50 points. A subsequent blinded recalculation, to ensure the accuracy of the ISS, did not show any statistical significance when compared to the on-site documented ISS (median (IQR) ISS 25 (20–38) points, *p* = 0.382). The median (IQR) NISS was 36 (24–57) points. Median (IQR) RISC II was calculated as 65 (25–88) %. In *n* = 85 (63%) of the patients, we found an Abbreviated Injury Scale (AIS) for the head ≥4 points with a median (IQR) preclinical Glasgow Coma Scale (GCS_preclincal_) of 3 (3–8) points. The median (IQR) GCS_ER_ when admitted to our trauma bay was 3 (3–4) points. A total of 29.1% of the patients died during the initial treatment in the trauma bay. Of those patients who survived the very early phase after admission, *n* = 95 were admitted to the intensive care unit and the median (IQR) length of stay was 2 (0–4) days, with 2 (1–4) days of ventilation.

Contrary to the preclinical assumption of a traumatic cause of the admission, in 25 patients (18.6%), an internal cause (e.g., syncopal fall from a standing position) was the cause of the hospitalization and death.

**Figure 2:**
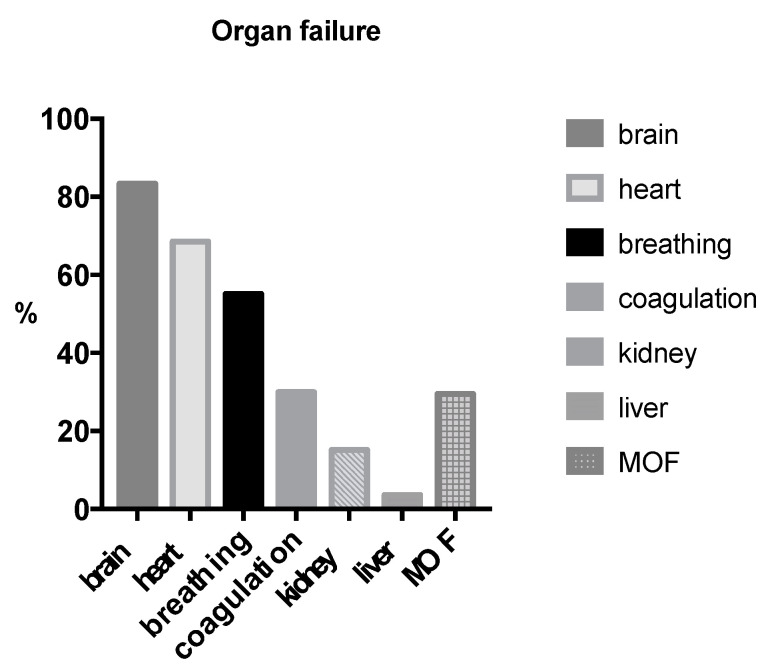
Percentage of post-traumatic organ failure in non-survivors. Brain (83.3%), heart (68.5%), breathing (55.1%) failure, coagulation (29.9%), kidney (15.1%) and liver (3.6%) failure, 29.5% multi-organ failure (MOF).

Table 2 and Figure 2 show preclinical and clinical mortality relevant risk factors and therapy decisions for the *n* = 135 deceased patients. In total, 91.5% of these patients were initially admitted to our hospital. Owing to cardiac arrest, preclinical cardiopulmonary resuscitation (CPR) was necessary in *n* = 39 (29.1%), and CPR was performed in the trauma bay (during the initial in-hospital treatment) in 32.1% of the patients. In total, 17.9% received massive blood transfusion (defined as transfusion of 10 units of packed red blood cells within 24 hours), and for 11.9% an emergency laparotomy had to be performed. Meanwhile, 30.4% of patients with severe TBI had to undergo emergency craniectomy. Emergency chest tubing was performed in 16.2%.

Patients admitted to the intensive care unit (ICU) most frequently suffered from post-traumatic organ failure of the central nervous system (83.3%), followed by heart (68.5%) and lung failure (55.1%). Finally, 88 patients suffered from multi-organ failure (MOF) (Figure 2).

The most interesting result of the factors influencing the mortality and therapy of these deceased seriously injured patients is that in the vast majority (64.9%) of cases with an uncertain prognosis, a ‘therapia minima’ (a limitation of therapy) was decided upon. This was determined mainly by consensus (64.9%) with the relatives and in accordance with the presumed will of the patient. A legally binding patient’s decree was available for 12.7% of the deceased patients.

## 4. Discussion

A total of 135 (6.2%) patients died between 2014 and 2016, after admission via our trauma bay in the hospital. In all cases, the patients were treated according to standardized algorithms [24,25,26,27,28]. The median ISS and mortality risk (RISC II) of the cohort was high. The leading causes of accident in our cohort (falls and traffic accidents) correspond to the available epidemiological data on severe multiple trauma [2,29,30]. The large proportion of falls from a height of less than 3 m is notable. According to the current and expected demographic development, an increasing number and clinical significance of these cases can be expected. As mentioned above, this affects a high number of patients with severe TBI. In these cases, especially if the patient is older, therapy is limited (‘therapia minima’) during intensive medical care with an uncertain prognosis in the presence of a patient decree, but mainly with the consent of the patient’s relatives. The relevance of these documents and the decision on second surgical interventions and intensive care must be carefully examined. They are particularly susceptible to misinterpretation in acute care as opposed to the chronic degradation process [6,7]. By means of a written advance directive, patients can stipulate as a precaution that certain medical measures are to be carried out or omitted if they can no longer decide for themselves. This ensures that the patient’s will is implemented even if it can no longer be expressed in the current situation. If there is no patient decree, or if the provisions in a patient decree are too vague or general, the representative decides together with the physician on the treatment to be given, based on the presumed will of the patient. If—in the case of decisions with particularly serious consequences—the representative and the attending physician cannot agree, the representative must obtain the consent of the responsible court [7].

Up to now, only the existence of a patient decree has been documented in the available registry data. However, a much larger part of the therapy limitations was based on the joint decision between physicians and the patient’s relatives, following the supposed will of the patients. The significance of therapy limitations in existing patient decrees is relevant and might result in a misinterpretation of the results showing a high mortality. Trauma surgery, with its high proportion of geriatric patients—which will continue to increase in the coming years—is often confronted with the problem of extending the therapy to seriously injured patients. For these patients, the existence of a living will, or precautionary power of attorney is a very valuable support for the medical decision. However, the question of whether a living will and/or a power of attorney is available often only arises during the course of treatment if serious complications have occurred. If there is no concretely formulated patient’s decree, the medical staff must determine the possible will of the patient concerned in discussions with, e.g., children, spouses or relatives and, if necessary, arrange for the establishment of legal care—a process that can be arduous for all parties involved and can be accompanied by a delayed implementation of the patient’s presumed will. The confrontation of relatives with decisions on future intensive supportive therapy is also not without problems and is very culture-specific, especially with regard to which limitations of quality of life are considered acceptable. Hack et al. determined the distribution of living wills, powers of attorney and legal care in the accident surgery-geriatric patient population of a university hospital in 2016. In their geriatric trauma patient collective, 63% of the patients had a living will, power of attorney, legal care or several of the documents mentioned [6]. The early involvement with this topic—if necessary, already during the initial contact with the patient in the emergency room—can avoid not only delays but also uncertainties in the decision-making process. A common problem, however, is that when a patient is admitted, there is no information about the existence of a patient’ decree, for example in the case of older patients with severe TBI admitted to the trauma bay without accompanying relatives. Information on patient decree and living wills is important core data in the (inpatient) treatment of geriatric patients; a clear documentation that can be viewed quickly and easily by doctors and nursing staff should be guaranteed accordingly. We have taken our results as an opportunity to integrate the query regarding patient’s decree and power of attorney (including the telephone numbers of contact persons) in the emergency admission form. The uniform documentation makes it easier to get a quick and easy overview of the necessary information when creating the patient chart and subsequently to implement the living will. The consideration of such documents is a decisive part of the treatment quality of geriatric patients. Finally, it should be pointed out here that living wills are not instruments of self-determination for elderly people alone. Serious accidents in particular naturally affect younger people.

According to our analysis, one third of the deceased patients were preclinically resuscitated. In more than 30% of cases, CPR was continued or performed after admission in the trauma bay. MOF remains a significant cause of morbidity after trauma resuscitation. If ongoing resuscitation is necessary in the emergency room after the preclinical beginning of resuscitation, the survival rate is known to be very low [31,32,33]. In addition, a relevant number of the deceased patients retrospectively showed an internal medical cause of death, which also influences mortality, depending on the severity of the injury. In many cases, syncope leads to falls at low altitude; often, cardiac patients belong to the group of multimorbid patients and are on permanent pre-existing medication, including a high percentage of patients treated with anticoagulant medication. In our study, we recognized that these patients are often misdirected via the trauma TC, especially if the nature of their fall is initially unclear. The reason for this is, among other things, the highly standardized treatment of severe injuries in trauma surgery, which has been established for decades, for example by ATLS^®^ [5]. This shows once again that interdisciplinary emergency admissions are of the highest relevance, especially in view of an increasingly ageing society. By immediately involving all appropriate disciplines, a relevant delay in determining the current leading diagnosis, such as myocardial infarction, and in initiating any necessary therapy (e.g., cardiac catheterization) can be avoided.

Unsafe healthcare delivery results in millions of patients worldwide suffering from potentially preventable harm or death. A Swedish study estimated the prevalence of preventable adverse events as high as 8.6% during hospital care; according to this, the identification of patient safety problems and provision of background data and information all aim to improve patient safety [11]. For this purpose, a registry-based retrospective mortality analysis as performed here is a useful tool for internal quality control. One of the main reasons for the high quality of the available registry data is certainly the implementation of a documentation assistant. This approach achieves a high documentation quality and subsequent analysis capability with low personnel and economic expenditure. After reviewing the available registry data, it can be concluded that of the 135 deaths, a high proportion of our patients showed a high severity of injury accompanied by a vital threat; nevertheless, the values suggested by the ISS should be interpreted with caution. The weakness of this score is reasonable in the underestimation of the patient with more than three injured body regions, in severe craniocerebral trauma with more than one injury location and morphology, and with multiple limb injuries [34]. The NISS tries to compensate for these weaknesses by taking into account additional clinical data, age and severity of the TBI, and therefore the NISS might be more appropriate in this patient group. However, despite various weaknesses, the ISS has been the most frequently used injury score since its initial description by Baker [20]. Nevertheless, we did not find any preventable deaths due to the interventions that were performed on the basis of the injury pattern. The main reason for this is a high proportion of severe TBIs with a devastating prognosis. After reviewing the available data, these patients died of brain damage following a mass hemorrhage or brain edema, despite emergency damage control, such as intracranial pressure monitoring (ICP) or hemicraniectomy. Patients with focal brain injuries, especially subdural hematomas, generally have a higher mortality than patients who have diffuse brain injuries [35]. The older the patient, the worse the compensatory capacity; the longer the unconsciousness and GCS, the lower the chance of survival or the chance to live on without defects [36,37,38]. As seen in our study, a high rate of older patients suffered from severe TBI with low GCS, which can explain the higher rate of mortality.

## 5. Conclusions

Most deaths were clearly related to the severity of the traumatic brain injury or other injuries with severe hemorrhagic shock. However, a significant number of deaths (71.6%) were not entirely based on medical reasons, but on a self-determined or otherwise implied limitation of treatment; this changes the percentage of survivors for ethical rather than medical reasons. Trauma surgery, with its high proportion of geriatric patients, is often faced with the problem of expanding the treatment of seriously injured patients. In this context, patient decrees or the presumed will of the patient are important support for medical decisions and must be taken into account in documentation and data collection. The establishment of a regular registry-supported mortality analysis can be an adequate tool for quality management in trauma surgery.

## Figures and Tables

**Table 1:** Basic patient data.

	Median	(IQR)		%
Age (y)	69	(38–83)	Sex (male)	71.1
ISS (pts)	25	(17–34)	AIS_head_ ≥4	41.0
ISS_recalculated_ (pts)	25	(20–38)	ISS ≥16	84.4
NISS (pts)	36	(24–57)	ISS ≥25	71.1
RISC II Score (%)	65	(29–88)	ISS ≥50	16.3
AIS_head_ (pts)	4	(2–5)		
AIS_face/neck_ (pts)	0	(0–0)	Internal cause of death	18.7
AIS_thorax_ (pts)	0	(0–3)		
AIS_abdomen_ (pts)	0	(0–0)		
AIS_extremities_ (pts)	0	(0–2)		
AIS_soft tissue_ (pts)	0	(0–1)		
GCS_preclinical_ (pts)	3	(3–8)		
GCS_ER_ (pts)	3	(3–4)		
ICU (d)	2	(0–4)		
ETI (d)	2	(1–4)		

Abbreviations: IQR: interquartile range, y: years, pts: points, d: days, AIS: Abbreviated Injury Scale, ISS: Injury Severity Score, GCS: Glasgow Coma Scale, ER: emergency room, ICU: intensive care unit, ETI: endotracheal intubation.

**Table 2:** Preclinical and clinical mortality-relevant therapy decisions (*n* = 135).

Treatment	%	Damage Control	%
Preclinical CPR	29.1	Massive blood transfusion	17.9
ETI	46.9		
Chest tubing	5.3	Chest tubing	16.2
TXA	11.4	ICP	26.9
		craniotomy	30.4
Primary admission	91.9	secondary	4.5
		Laparotomy/Packing	11.9
Clinical CPR	32.1	ECMO	4.5
KAT	46.2	Spine surgery	1.5
ETI	31.1		
Therapy limitation	71.6		
Patient decree	12.7		
In consensus with relevant	64.9		

Abbreviations: ETI: endotracheal intubation, CPR: cardiopulmonary reanimation, TXA: tranexamic acid, KAT: catecholamine, ICP: intracerebral pressure monitoring, ECMO: extracorporeal membrane oxygenation.
